# Comparative Evaluation of Efficacy of Chitosan and Chlorhexidine Mouthwash in Plaque Control and Gingivitis: An Observational Study

**DOI:** 10.7759/cureus.70810

**Published:** 2024-10-04

**Authors:** Kratee Sharma, Ellora Madan, Anubha Nirwal, Zaby Fatima

**Affiliations:** 1 Department of Periodontics, Kothiwal Dental College and Research Centre, Moradabad, IND

**Keywords:** gingivitis, mouthwash, plaque, retrospective, stain

## Abstract

Introduction: Mouth rinse is a highly effective chemical solution for managing plaque and gingivitis. This study evaluated and compared the efficacy of chitosan (CH)-rich mouthwash with standard chlorhexidine (CHX) mouthwash in reducing gingivitis, plaque, halitosis, and stains.

Materials and methods: This retrospective, observational study was conducted on the clinical case records of 60 patients who were treated with CH mouthwash (formulated and dispensed from the department) as group 1 and CHX mouthwash as group 2 for at least three months. Indices such as the plaque index (PI), gingival index (GI), stain index (SI), and halitosis index (HI) were obtained at baseline (T0) and the end of three months of mouthwash use (T1) from the case records. The data were subjected to statistical analysis.

Results: Both groups effectively reduced all indices from T0 to T1; however, group 1 was more effective than group 2 in reducing gingival, plaque, and stain scores. The results were statistically significant (p<0.05) for all indices, and both groups were equally effective in reducing halitosis. Male sex showed a significant positive correlation with PI, GI, and SI, whereas female sex showed a positive correlation with PI and HI.

Conclusion: Based on the findings of the present study, it was concluded that the CH mouthwash was more effective in reducing plaque, gingival, and staining scores than the CHX mouthwash. Both types of mouthwash were equally effective in reducing halitosis scores.

## Introduction

Dental plaque is the primary contributor to gingivitis and inflammatory periodontal diseases; therefore, it must be effectively removed using mechanical and chemical plaque control methods [1]. Chlorhexidine digluconate (CHX) is acknowledged as the gold standard for chemical plaque control. The use of CHX mouthwash at concentrations ranging from 0.1% to 0.2% exhibits considerable anti-plaque properties when administered daily over a duration of two weeks without the inclusion of mechanical cleaning and serves effectively as a long-term supplement to oral hygiene practices at intervals of four to six weeks and six months [2,3].

The application of CHX as a mouthwash is not devoid of undesirable effects, with some of the most frequently encountered being xerostomia (dry mouth), alterations in taste perception (hypogeusia), particularly for salty and bitter flavors, and the occurrence of discolored or coated tongue. Notwithstanding its anti-plaque efficacy, there have been reports indicating that the 0.12% CHX mouthwash may also contribute to an increase in calculus formation. Nonetheless, the most concerning consequence that discourages patients from using CHX mouthwash is tooth discoloration [4]. This phenomenon is prevalent when usage exceeds several weeks owing to non-enzymatic browning (Maillard reaction) and the generation of pigmented metal sulfide compounds within the pellicle. It is also recommended for limited-duration use only; typically, 2-4 weeks, with licensing in the UK permitting utilization for a maximum of 30 days [5].

Therefore, several investigations on natural compounds, such as chitosan (CH), have been prompted by the unfavorable side effects of CHX [6]. CH, an organic polysaccharide biopolymer called CH, is produced by the alkaline deacetylation of chitin [7]. CH's many health benefits have drawn much interest as a chemical agent for mouthwashes [8]. In a comprehensive systematic review conducted by Pandiyan et al. [9], only three investigations were ultimately incorporated into their analysis, assessing the effectiveness of CH in conjunction with CHX; these studies were executed over a brief timeframe [9]. Due to limited research on this topic, the present study was conducted with the aim of assessing the efficacy of CH and CHX mouthwashes as anti-plaque agents.

## Materials and methods

Study design

This retrospective, observational study was conducted in the Department of Periodontology, Kothiwal Dental College and Research Centre, Moradabad, using the archival records of patients who visited the department between January 2021 and December 2023. Written informed consent was obtained from the department’s protocol to use the patients' records for research purposes and to maintain confidentiality. The procedure followed in the study was in accordance with the ethical standards of the Institutional Ethics and Review Board (KDCRC/IERB/02/2021/16) and the Helsinki Declaration of 1975, as revised in 2000.

Sample size estimation

The G-power analysis in the present study estimated a total sample of 60 (30 in each group) at 20% beta error and 5% alpha error. The marginal effect size between the efficacy of the two groups on plaque index was 0.65 [6].

Patient selection 

This study included the case history records of systemically healthy patients between the ages of 20 and 45 years with gingivitis as diagnosed by clinical examination using a gingival index (GI) by Loe and Silness [10] with scores ≥ 1, who were prescribed CH or CHX mouthwash for three months, patients did not have a history of periodontal therapy within the preceding six months, and who had complete clinical records. Incomplete records, records with a history of patients using antibiotics, steroids, or hormonal therapy within the last three months of their visit, multiple missing teeth, periodontally compromised teeth, medically compromised patients, and individuals with disease severity requiring periodontal therapy other than scaling and root planning were excluded from the study.

Methodology

The selected records (n=60) were divided into two groups: group 1, where CH (formulated and dispensed from the department) was used for gingivitis (n=30), and group 2, where CHX mouthwash was used (n=30). CH mouthwash was prepared according to the instructions of Sano et al. [11]. Preparations were made at the Department of Pharmacy, Institute of Foreign Trade and Management (IFTM) University, Moradabad.

As indicated in the case records, after the procedure of thorough oral prophylaxis, the patients in group 1 used 0.5% CH mouthwash, 10 ml for 60 seconds, twice per day after meals for a period of three months. No changes were made to the patient’s oral hygiene methods. The patients in Group 2 used 0.2% CHX mouthwash following the same instructions and methods as Group 1. The following indices were assessed to evaluate the efficacy of two-mouth rinses: plaque index (PI) by Turesky et al., who modified Quigley-Hein PI [12], GI by Loe and Silness [10], stain index (SI) by Lobene [13], and halitosis index (HI) by Rosenberg and McCulloch [14]. The baseline indices (PI, GI, SI, and HI) were obtained from the patient’s case history records (T0). After three months of mouthwash use, the indices were re-recorded (T1).

Statistical analysis

The data were entered into Microsoft Excel and analyzed using IBM Corp. Released 2013. IBM SPSS Statistics for Windows, Version 22.0. Armonk, NY: IBM Corp. Categorical data, such as sex and brushing frequency, were presented as frequencies and percentages and analyzed using the chi-square test. Continuous data, including age and clinical parameters (PI, GI, SI, and HI), are presented as means and standard deviations and analyzed using the t-test for group comparisons. Paired t-tests were used to assess the changes between pre- and post-treatment values within the groups. Differences between groups and over time were evaluated using mixed-model analysis of variance (ANOVA). The threshold for statistical significance was set at a p-value of 0.05.

## Results

Table 1 presents the demographic characteristics of the patients in groups 1 (n=30) and 2 (n=30).

**Table 1:** Descriptive analysis of the sample. *Independent t test, ** chi-square test, p>0.05: non-significant (NS).

Parameters	Group 1 (n=30)	Group 2 (n=30)	p-value	Effect size
*Age in years (Mean±SD)	34.43 ± 7.05	32.83 ± 6.05	0.349 (NS)	0.24
**Gender n (%)				
Male	18 (60%)	15 (50%)	0.301 (NS)	0.19
Female	12 (40%)	15 (50%)		
**Brushing n (%)				
Once	21 (70%)	24 (80%)	0.371 (NS)	0.16
Twice	9 (30%)	6 (20%)		

The mean age of the patients in group 1 was 34.43 ± 7.05 years, while in group 2, it was 32.83 ± 6.05 years, with a p-value of 0.349 and a small effect size of 0.24, indicating no statistically significant age difference between the groups. Sex distribution also showed no statistically significant difference (p=0.301, effect size 0.19), with males comprising 60% of group 1 and 50% of group 2. Brushing frequency, predominantly once daily, showed no statistically significant difference between groups (p=0.371, effect size 0.16).

Table 2 represents a comparison of clinical parameters between the groups using an independent T-test.

**Table 2:** Comparison of improvement in the indices after treatment in both study groups using an Independent T test. *p<0.05: Significant, MD: Mean difference (T1-T0), SD: Standard deviation.

Parameter	Group 1 (n=30) MD±SD	Group 2 (n=30) MD±SD	p-value	Effect size
Plaque index	0.66 ± 0.6	0.28 ± 0.3	0.003*	0.81
Gingival index	1.45 ± 0.19	1.32 ± 0.18	0.013*	0.66
Stain index	0.41 ± 0.37	0.15 ± 0.36	0.007*	0.72
Halitosis index	1.37 ± 0.45	1 ± 0.44	0.002*	0.82

The improvement in PI was significantly higher in group 1 (0.66 ± 0.6) than in group 2 (0.28 ± 0.3, p=0.003, effect size 0.81), indicating a large effect. Similarly, the improvement in GI was also significantly higher in group 1 (1.45 ± 0.19) than in group 2 (1.32 ± 0.18, p=0.013, effect size 0.66). The improvement in both the SI and HI was significantly greater in group 1, with p-values of 0.007 and 0.002, respectively, and large effect sizes (0.72 and 0.82, respectively). This showed that the CH mouthwash was more effective than the CHX mouthwash in gingivitis.

Table 3 shows the results of the comparison of the indices between the groups over time.

**Table 3:** Mixed-model ANOVA for comparison of both groups at baseline (T0) and after third month (T1). PI: Plaque index, GI: Gingival index, SI: Stain index, HI: halitosis index, *p < 0.05: Significant, RM: Repeated measures.

Indices Group 1- Group 2		Mean square	F value	p-value	η^2^
PI	T0 vs. T1	6.63	59.33	0.001*	0.29
	Group	2.68	25.52	0.001*	0.12
	RM factor x group	1.09	9.74	0.003*	0.05
GI	T0 vs. T1	57.51	3282.32	0.001*	0.96
	Group	0.13	8.64	0.005*	0
	RM factor x group	0.11	6.55	0.013*	0
SI	T0 vs. T1	2.35	35.6	0.001*	0.21
	Group	0.03	0.39	0.033*	0
	RM factor x group	0.52	7.87	0.007*	0.05
HI	T0 vs. T1	42.01	424.97	0.001*	0.77
	Group	0.01	0.09	0.769	0
	RM factor x group	1.01	10.2	0.002*	0.02

The analysis revealed significant reductions in PI, GI, SI, and HI over time, with large effect sizes (η² = 0.29 for PI, 0.96 for GI, 0.21 for SI, and 0.77 for HI). For PI, there were significant differences between the groups (p < 0.001, η² = 0.12) and a significant time-group interaction (p = 0.003, η² = 0.05). The GI showed a strong time effect (p < 0.001, η² = 0.96) and a small group effect (p = 0.005). The SI displayed significant time (p < 0.001, η² = 0.21) and interaction effects (p = 0.007, η² = 0.05), while group differences were significant (p = 0.033). HI showed a significant interaction effect (p = 0.002, η² = 0.02), with no group effect (p = 0.769). This showed that both mouthwashes were particularly effective in reducing halitosis; however, the CH mouthwash was more effective in plaque control, gingivitis, and stain reduction.

Table 4 represents the correlation analysis (r) and significance (p-value) for the clinical indices between the groups.

**Table 4:** Correlation analysis using point biserial correlation test for different clinical parameters. *p<0.05: Significant, Very weak correlation: 0.0<∣r∣<0.20; Weak: 0.2≤∣r∣<0.4; Moderate: 0.4≤∣r∣<0.6; Strong: 0.6≤∣r∣<0.8; Very Strong: 0.8≤∣r∣≤1.

Parameters	Correlation (r value), significance (p value)	Plaque index	Gingival index	Stain index	Halitosis index
Group 1 vs. group 2	r	0.38	0.32	0.35	0.39
	p	0.003*	0.013*	0.007*	0.002*
Males in both groups	r	0.38	0.4	0.4	0.34
	p	0.033*	0.025*	0.023*	0.54
Females in both groups	r	0.45	0.26	0.32	0.45
	p	0.016*	0.174	0.102	0.016*
Brushing once in both groups	r	0.45	0.54	0.35	0.47
	p	0.002*	0.001*	0.019*	0.001*
Brushing twice in both groups	r	0.31	-0.18	0.34	0.22
	p	0.243	0.493	0.202	0.405

Significant positive correlations were found for the PI (r=0.38, p=0.003), GI (r=0.32, p=0.013), SI (r=0.35, p=0.007), and HI (r=0.39, p=0.002). In males, all indices except HI were significantly correlated, whereas in females, PI and HI showed significant correlations. Those brushing once daily showed strong correlations across all indices, whereas brushing twice had no significant correlations. In simple terms, the study found that brushing once daily linked these indices more closely, whereas brushing twice daily did not show the same connection. Additionally, the strength of these relationships varies between men and women. In males, PI, GI, and SI tended to increase together, but HI did not show the same relationship, whereas in females, PI and HI increased together.

## Discussion

The results of the present study indicated that both CH and CHX mouthwash were effective in reducing the PI, GI, SI, and HI of the patients from baseline to three months of usage. This is in accordance with the findings of previous studies [6-9]. CH exhibits antibacterial, antioxidant, and mucoadhesive properties. It exhibits an anti-adhesion capacity that triggers alterations in the bacterial surface architecture, modifies the expression levels of bacterial surface ligands, and enhances adsorption to hydroxyapatite crystals located on dental surfaces. These characteristics elucidate the bactericidal and bacteriostatic effects attributed to CH [11,15]. A previous study investigated the bactericidal efficacy of CH through both in vivo and in vitro assessments and concluded that the antibacterial capabilities of CH were comparable to those of commercially available mouthwashes [16]. It is plausible that the antibacterial properties of CH result from an interplay between mechanisms involving binding to bacterial cell surfaces and those pertaining to the interaction with DNA.

As an antiseptic oral rinse, CHX exhibits antimicrobial properties against a variety of pathogens, including bacteria, fungi, and viruses, implicated in several oral disorders. In vitro studies have indicated that the antibacterial mechanisms of CHX are linked to modifications in the cell membrane permeability. At elevated concentrations (>0.1%), CHX induces the efflux of crucial intracellular constituents, leading to a bactericidal effect characterized by cell lysis and subsequent cell death [17]. CHX has the potential to provide certain clinical advantages in the management of gingivitis, as evidenced by a systematic review that revealed that a regimen of daily rinsing with 0.2% CHX over a span of 4 to 6 weeks led to a reduction in clinical manifestations across multiple studies [18]. Nevertheless, the recent consensus guidelines issued by the European Federation of Periodontology (EFP) clearly indicate that such antiseptic agents should be implemented as a supplementary measure alongside mechanical tooth brushing and interdental cleaning.

The results of the present study indicated that the CH mouthwash was more effective than the CHX mouthwash in gingivitis. These findings may be explained by the advantageous bioadhesive characteristics of CH and its effective adherence to oral mucosal surfaces. This finding was in accordance with that of Uraz et al. [19]. However, a previous study by Mhaske et al. [20] and Vilasan et al. [6] reported no statistically significant differences in both groups regarding the reduction in PI, GI, and HI. These contradictory results might be due to the fact that both studies were conducted for a short duration of four days [20] and a small sample size [6]. However, both studies reported synergistic effects of CH and CHX mouthwashes when used in combination.

The present study revealed that CH was more effective in reducing stains than CHX mouthwash. This might be because when CHX mouthwash usage persists beyond several weeks, staining occurs, which is attributed to non-enzymatic browning (Maillard reaction) and the resultant formation of pigmented metal sulfide within the pellicle. Consequently, this may facilitate binding interactions between tin and iron with dietary aldehydes and ketones, thereby promoting the deposition of food constituents on dental surfaces [17]. Additional adverse effects linked to the utilization of CHX mouthwash include dryness of the mouth, modifications in taste perception, the presence of a discolored or coated tongue, sensations of burning, and desquamation of the oral mucosa [17]. Therefore, CHX mouthwash is usually not recommended for long-term use, such as during orthodontic treatment, which lasts for approximately three years. These side effects were not observed with the CH mouthwash and, hence, are recommended for long-term use.

The results of this study revealed that both groups were equally effective in reducing halitosis. Owing to the lack of previous studies, this finding could not be supported by previous studies. The most probable reason for the above finding could be that both CH and CHX exhibit potent antimicrobial characteristics that assist in diminishing the population of oral microorganisms responsible for the synthesis of volatile sulfur compounds (VSCs). These compounds are principal contributors to halitosis. By curtailing these microorganisms, both agents directly mitigate the generation of compounds responsible for the malodor.

Clinical implications

CHX mouthwash, while effective, is associated with side effects such as staining of teeth, altered taste sensation, and mucosal irritation. CH showed better efficacy in reducing gingivitis; with fewer side effects, it could be a safer and more patient-friendly alternative. CH is obtained from chitin, a naturally occurring polymer present in the exoskeletons of crustaceans. Its remarkable biocompatibility renders it an advantageous choice for individuals who favor natural or biocompatible substances over synthetic agents, such as CHX.

Limitations

The major limitation of the current study was its retrospective design, which limited the generalizability of the study. Historical data may exhibit incompleteness or inconsistencies attributable to inaccuracies in documentation practices or variations in the methodologies employed for data collection. However, this was carefully addressed in the present study by eliminating records with missing or incomplete data. Moreover, the synergistic effects of CH and CHX were not evaluated in the present study. Further randomized controlled trials are needed to confirm the long-term effects and comparative benefits.

## Conclusions

Based on the findings of the present study, it was concluded that the CH mouthwash was more effective than the CHX mouthwash in reducing plaque, gingival, and staining scores. Both types of mouthwash were equally effective in reducing the halitosis scores. Both groups showed significant reductions in PI, GI, SI, and HI over time with large effect sizes. Among all the indices, GI showed a strong time effect. Male sex showed a significant positive correlation with PI, GI, and SI, whereas female sex showed a positive correlation with PI and HI.
